# Complete Genome Sequences of IncI1 Plasmids Carrying Extended-Spectrum β-Lactamase Genes

**DOI:** 10.1128/genomeA.00859-14

**Published:** 2014-08-28

**Authors:** Michael S. M. Brouwer, Alex Bossers, Frank Harders, Alieda van Essen-Zandbergen, Dik J. Mevius, Hilde E. Smith

**Affiliations:** Central Veterinary Institute, Wageningen UR, Lelystad, The Netherlands

## Abstract

Extended spectrum beta-lactamases (ESBLs) confer resistance to clinically relevant antibiotics. Often, the resistance genes are carried by conjugative plasmids which are responsible for dissemination. Five IncI1 plasmids carrying ESBLs from commensal and clinical *Escherichia coli* isolates were completely sequenced and annotated along with a non-ESBL carrying IncI1 plasmid.

## GENOME ANNOUNCEMENT

Antibiotic resistance is a continuously growing concern for treatment of bacterial infections in human and veterinary medicine. Resistance genes are often carried by mobile genetic elements such as plasmids through which resistance is spread within an environment (1). Extended spectrum beta-lactamases (ESBLs) are multidrug-resistant proteins which can hydrolyze a variety of clinically relevant antibiotics such as penicillins and cephalosporins. Frequently, ESBLs are carried by plasmids of the incompatibility group I1 which are present in *Escherichia coli* from diverse environments such as feces from animals in bio-industry and wildlife, human clinical samples, and food stuffs intended for human consumption (2–6). In general, IncI1 plasmids measure 90 to 120 kb and carry various antibiotic-resistant genes and plasmid addiction systems (7, 8).

Five IncI1 plasmids were isolated from cefotaxime resistant isolates and one from a cefotaxime susceptible isolate. The presence and types of ESBLs were determined by microarray and target specific PCR and sequencing (2), and plasmid sequence types (ST) were determined by pMLST (9). Plasmids pESBL-12 (ST37) and pESBL-117 (ST36), respectively, carry *bla*_CTX-M-15_ and *bla*_TEM-52_, whereas pESBL-283 (ST7), pESBL-305 (ST3), and pESBL-315 (incomplete ST) carry *bla*_CTX-M-1_ and pE17.16 (ST24) carries no ESBLs. Plasmids pESBL-12 and pESBL-117 were present in *E. coli* cultured from human urine samples, pESBL-283 from pig feces, and pESBL-305, pESBL-315, and pE17.16 from chicken cecum content. These plasmids were selected for sequencing as they represent a broad selection of STs isolated from diverse sources and they carry various ESBLs.

Next-generation whole-genome sequencing of the plasmids was performed by shotgun XL pyrosequencing (454-Roche XL sequencer). *De novo* assembly of the reads was performed using Newbler v2.5.3. Scaffolds were built by custom scripts using synteny of contigs determined by BLAT v34 (10) mapping on reference sequence R64 (7). These scaffolds (artificial chromosomes), which also included any non-homologous contigs with reference R64, were compared using customized Nucmer (MUMmer v3.07 [11]) scripts allowing sequence comparison and curation in Artemis and ACT (12, 13). PCR and subsequent Sanger sequencing (ABI-Prism GA3130) were performed to close any gaps between the scaffolded contigs. Final annotation of the plasmids was performed with the NCBI Prokaryotic annotation pipeline (http://www.ncbi.nlm.nih.gov/genome/annotation_prok/) and was manually curated.

The plasmids sequenced here range in size between 89,503 and 110,137 bp, the G+C content of ranges between 49.5% and 51.4% and between 101 and 129 coding sequences (CDSs) were predicted per plasmid. In addition to beta-lactamases, which are present on all plasmids except additional plasmid pE17.16, various resistance genes are present. Resistance to aminoglycosides, sulfonamide, and trimethoprim is carried by pESBL-283, pESBL-305, and pESBL-315, whereas pESBL-12 carries resistance to aminoglycosides and pE17.16 carriess resistance to aminoglycosides and sulfonamide.

Each of the plasmids carries the pndCA plasmid addiction system in which translation of the toxin mRNA is prevented by an unstable sRNA (7, 8, 14). In addition, pESBL-117 carries the virulence associated ribonuclease VapBC system (15) and pESBL-283, pESBL-305, pESBL315, and pE17.16 carry the ribosome-dependent mRNA cleavage system RelBE (16).

These nucleotide sequences confirm that there is high conservation in the backbone of IncI1 plasmids.

### Nucleotide sequence accession numbers.

Genome sequences have been submitted to GenBank under the accession numbers listed in Table 1.

**TABLE 1:** Plasmid sequence accession numbers

Plasmid	Accession number
pE17.16	CP008733
pESBL-117	CP008734
pESBL-12	CP008735
pESBL-283	CP008736
pESBL-305	CP008737
pESBL-315	CP008738
